# Monitoring and kinetic analysis of the molecular interactions by which a repressor protein, PhaR, binds to target DNAs and poly[(*R*)-3-hydroxybutyrate]

**DOI:** 10.1186/2191-0855-3-6

**Published:** 2013-01-27

**Authors:** Miwa Yamada, Shuntaro Takahashi, Yoshio Okahata, Yoshiharu Doi, Keiji Numata

**Affiliations:** 1 Enzyme Research Team, RIKEN Biomass Engineering Program, RIKEN, 2-1, Hirosawa, Wako-shi, Saitama, 351-0198, Japan; 2 Department of Biomolecular Engineering, Tokyo Institute of Technology, 4259 Nagatsuta, Midori-ku, Yokohama, 226-8501, Japan; 3 Research Cluster for Innovation, RIKEN, 2-1, Hirosawa, Wako-shi, Saitama, 351-0198, Japan; 4 Present address: Department of Biological Chemistry and Food Science, Faculty of Agriculture, Iwate University, 3-18-8 Ueda, Morioka, 020-8550, Japan; 5 Present address: Frontier Institute for Biomolecular Engineering Research (FIBER), Konan University, 7-1-20 Minatojima-minamimachi, Chuo-ku, Kobe, 650-0047, Japan

**Keywords:** Polyhydroxyalkanoate, Autoregulator protein PhaR, Kinetic analysis, *Ralstonia eutropha* H16

## Abstract

The repressor protein PhaR, which is a component of poly[(*R*)-3-hydroxybutyrate] granules, functions as a repressor of the gene expression of the phasin PhaP and of PhaR itself. We used a quartz crystal microbalance to investigate the binding behavior by which PhaR in *Ralstonia eutropha* H16 targets DNAs and amorphous poly[(*R*)-3-hydroxybutyrate] thin films. Binding rate constants, dissociation rate constants, and dissociation constants of the binding of PhaR to DNA and to amorphous poly[(*R*)-3-hydroxybutyrate] suggested that PhaR bind to both in a similar manner. On the basis of the binding rate constant values, we proposed that the *phaP* gene would be derepressed in harmony with the ratio of the concentration of the target DNA to the concentration of amorphous poly[(*R*)-3-hydroxybutyrate] at the start of poly[(*R*)-3-hydroxybutyrate] synthesis in *R. eutropha* H16.

## Introduction

Polyhydroxyalkanoate (PHA), an eco-friendly and biodegradable polyester, is synthesized by a variety of bacteria, as their intracellular storage material for carbon and energy (Doi et al. 1995; Steinbuchel and Fuchtenbusch 1998; Sudesh et al. 2000). In bacterial cells, PHA forms granules that are covered with a layer composed of proteins and phospholipids (Potter et al. 2002). The most abundant constituent of this layer is phasin (PhaP). The presence of PhaP on the surface of PHA granules contributes to the reduction in size of PHA granules as well as to the slight enhancement of PHA production (Kojima et al. 2006; Potter et al. 2002; Potter and Steinbuchel 2005). Recently, the ability of PhaP to bind to a hydrophobic surface was used to develop methods for protein purification, drug delivery, and tissue engineering applications in *in vitro* experiments (Backstrom et al. 2007; Banki et al. 2005; Wang et al. 2008). In the cells of microorganisms, a repressor protein PhaR regulates the expression of *phaP* and *phaR*. PhaR has also been reported to sense the presence of PHA and to interact with nascent PHA granules, resulting in the derepression of *phaP* expression (Potter et al. 2002; Potter and Steinbuchel 2005). The presence of genes homologous to PhaR and PhaP in the genomes of various PHA-producing bacteria suggests that a similar regulatory system by PhaR is likely to exist in PHA-producing bacteria (Eugenio et al. 2010; Kojima et al. 2006; Maehara et al. 2002; Yamada et al. 2007; Yamashita et al. 2006). This regulatory system of PHA production through *phaR* and *phaP* expression can be applied in a two-hybrid system for protein-protein interaction (Wang et al. 2011). Therefore, understanding of the regulatory system provides meaningful benefit to not only basic science but also applications in various fields such as industry and medicine.

In previous studies, the binding behaviors of PhaR to target DNA (including the promoter region of *phaP*) and to melt-crystallized thin films of poly[(*R*)-3-hydroxybutyrate] [cr-P(3HB)] were investigated using surface plasmon resonance (SPR) and quartz crystal microbalance (QCM) measurements (Yamada et al. 2007; Yamashita et al. 2006). However, kinetic parameters such as the binding rate constant (*k*_on_) and dissociation rate constant (*k*_off_) by which PhaR targets DNA and P(3HB) have not been determined thus far. These kinetics and stoichiometric analyses will contribute new insights into the behavior of PhaR in the regulatory system of *phaP* expression. In order to determine the precise kinetic parameters, we selected a multichannel QCM sensing system to monitor the binding reaction of PhaR from *Ralstonia eutropha* H16 to target DNAs (including the promoter regions of *phaP* and *phaR*) and thin films of amorphous P(3HB) [am-P(3HB)] derived from atactic P(3HB). This is because the P(3HB) native granule is composed of am-P(3HB). Recently, the regulatory system of PHA production through *phaR* and *phaP* expression has been applied in studies of protein-protein interaction, protein purification, drug delivery, and tissue engineering. The insights gained into this regulation mechanism in this study have the potential to improve applications in white biotechnology. We have determined kinetic parameters based on mass changes on the DNA-immobilized and am-P(3HB)-coated QCM oscillators, and discuss the binding behavior of PhaR with target DNA and am-P(3HB).

## Methods

### Expression and purification of autoregulator protein PhaR

**Figure 1:**
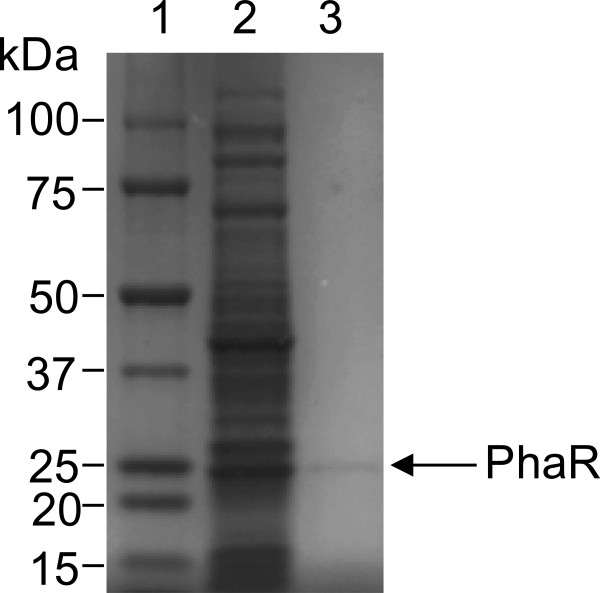
**Purification profiles of recombinant PhaR. **Soluble proteins were subjected to electrophoresis in an SDS (4-15%) polyacrylamide gradient gel and stained with CBB. Lane 1, molecular mass standard proteins; lane 2, soluble protein fraction of *E. coli* BL21 (DE3) harboring pET100/D-TOPO-PhaR_Re_.; lane 3, PhaR purified by nickel-nitrilotriacetic acid agarose (5 μg).

All chemical reagents were purchased from Wako Pure Chemicals (Osaka, Japan). The *phaR* gene from *R. eutropha* H16 was cloned using a TOPO TA cloning Kit (Invitrogen, Carlsbad, CA) with the forward primer 5′-CACCATGGCCACGACCAAAAAAGG-3′ and reverse primer 5′-TTACTTCTTGTCCGGCTGGT-3′. The resultant plasmid is referred to as pET100/D-TOPO-PhaR_Re_. The expression of the *phaR* gene was driven by the T5 promoter, which is inducible with isopropyl-α-d-thiogalactopyranoside (IPTG). The constructed plasmid was introduced into *Escherichia coli* BL21(DE3). Transformants were grown in 1200 mL of Luria-Bertani medium containing ampicillin (100 μg/mL) and kanamycin (50 μg/mL). They were cultivated at 30°C until the OD_600_ of the culture reached 0.5. After the addition of IPTG (final concentration of 1 mM), the transformants were grown for an additional 5 h. The cells were then harvested and washed with chilled buffer A (50 mM sodium phosphate (pH 8.0) containing 300 mM NaCl and 10 mM imidazole), and were suspended in 60 mL of the same buffer. The suspension was stored at −80°C until use. The suspension was thawed on ice and disrupted by sonic oscillation, also on ice. The cell debris was then removed by centrifugation at 15000 × *g* for 60 min at 4°C, and the supernatant was collected for purification. The experiments were carried out at 4°C throughout the purification steps. The crude extract was shaken gently with nickel-nitrilotriacetic acid agarose (Qiagen, Valencia, CA) for 1 h. The mixture was then poured into a column. The column was washed with buffer A containing 20 mM imidazole, and then the His-tagged protein was eluted with buffer A containing 250 mM imidazole. The eluates containing PhaR were dialyzed against 10 mM HEPES (pH 7.4) containing 150 mM NaCl and 3 mM EDTA and stored at −80°C. The protein concentration was determined using a Bio-Rad Protein Assay Kit (Bio-Rad, Hercules, CA) with bovine serum albumin as the standard. Proteins were separated by sodium dodecyl sulfate (SDS)-12.5% polyacrylamide gel electrophoresis (PAGE) and stained with Coomassie brilliant blue (CBB) R-250 (BioRad) as described by Laemmli (Figure 1).

### Calibration of 27-MHz QCM in aqueous solution

The QCM apparatus was an AFFINIX Q4 (Initium Co., Ltd., Tokyo, Japan) with 4 500-μL cells equipped with a 27-MHz QCM plate (8.7 mm diameter quartz plate and 5.7 mm^2^ area Au electrode) at the bottom of the cell and a stirring bar with a temperature control system (Takahashi et al. 2007; Takahashi et al. 2008). The relationship between mass and frequency changes in aqueous solutions when DNAs and/or proteins were immobilized onto the QCM was calibrated by comparing it against values in the air phase. One Hz of frequency represents a 0.10 ng cm^-2^ mass increase on the QCM plate. The noise level of the 27-MHz QCM was ±2 Hz in buffer solutions at 25°C, and the stability of the frequency was ±2 Hz for 1 h in buffer at 25°C.

### Preparation of the DNA-Immobilized QCM Oscillator

The structures of the biotinylated oligonucleotides used in this study are summarized in Table 1: they consisted of 5-biotinylated dsDNA (50 bp) containing a site recognized by PhaR dsDNA (*phaP* promoter region DNA and *phaR* promoter region DNA) and no-site dsDNA (control DNA). Oligonucleotide duplexes were formed by mixing a biotinylated strand and its complementary strand in a solution of 10 mM Tris–HCl (pH 7.8), 1 mM EDTA, and 200 mM NaCl, and then boiling for a few minutes, followed by cooling to room temperature over 1 h. These oligonucleotides were immobilized on a cleaned Au electrode of the QCM using biotin-avidin linkage according to the methods described in a previous paper (Okahata et al. 1998). The amount of immobilized DNA was maintained at 191 ng (0.55 – 0.02 pmol) cm^-2^, which corresponds to 1% coverage of the Au surface (5.7 mm^2^). This would allow sufficient space to accommodate the binding of a large enzyme molecule.

**Table 1:** **Kinetic parameters for the binding of PhaR to DNAs and P(3HB) on the 27-MHz QCM**^**a**^

**Targets**^**b**^	**DNA sequence**^**c**^	***k***_**on**_^**d**^**(10**^**-4**^ **M**^**-1**^ **s**^**-1**^**)**	***k***_**off**_^**c**^**(10**^**-3**^ **s**^**-1**^**)**	***K***_**d**_^***e***^**(10**^**-7**^ **M)**
Control DNA^f^	5′bio-TCGTTTAACGAGCCCGTATTTTCCCCTCTACCTTTTAGAGGACACCTAAC-3′	0.4	0.7	18
*phaP1* promoter^g^	5′bio-GGCGCATTTC**TTATTTGGTGCGCCGCAACAATTCCTATTTTA**GGGGCGCC-3′	6.0 ± 0.4	1.7 ± 0.4	3.2 ± 0.9
*phaR* promoter^f^	5′bio-TCACGCGTTTAGCCATAGCGG**GCGCGGTA**GACGAACAACAGCACGGCCGG-3′	0.5	0.9	18
am-P(3HB)^g^		7.0 ± 3.8	-^h^	-^h^

^a^ 10 mM HEPES buffer solution (pH 7.4) containing 150 mM NaCl and 0.002% Tween 20, 25°C. ^b^ 5′-biotinylated dsDNA immobilized on an avidin-covered QCM plate.

Amorphous-P(3HB) thin film was prepared by casting chloroform solution (1.0 wt%) of the isotactic P[(*R*), (*S*)-3-hydroxybutyrate]. ^c^ The bold type indicates the DNA binding site of PhaR revealed by DNase I footprinting (4). ^d^*k*_on_ and *k*_off_ were obtained from Eq. 4. ^e^*K*_d_ was calculated as *k*_off_/*k*_on_. ^f^ The data are from 2 independent experiments. ^g^ Amorphous P(3HB); the data are from 3 independent experiments. ^h^*K*_d_ value could not be calculated due to a negative *k*_off_ value.

### Preparation of P(3HB) thin films

QCM oscillators were washed with a freshly prepared Piranha solution of H_2_O_2_/H_2_SO_4_ (1/3 v/v) and were rinsed several times with Milli-Q water. (Caution: Piranha solution is very oxidative and dangerous, and direct contact should be avoided). Thin films of P(3HB) were prepared on the QCM oscillators by casting 300 μl of chloroform solutions (1.0–1.5 wt%) of the polymers on a spin-coater at 4000 rpm under dry air.

### Reactions in the DNA-immobilized or am-P(3HB) coated QCM oscillator

Enzyme reactions in a DNA-immobilized or am-P(3HB) coated QCM cell were performed with 500 μL of assay buffer (10 mM HEPES (pH 7.4), 150 mM NaCl, and 0.002% Tween 20). The frequency changes in response to the addition of enzymes were then followed over time. The solution was vigorously stirred to avoid any effects from the slow diffusion of the enzymes. The stirring did not affect the stability or magnitude of the frequency changes.

## Results

**Figure 2:**
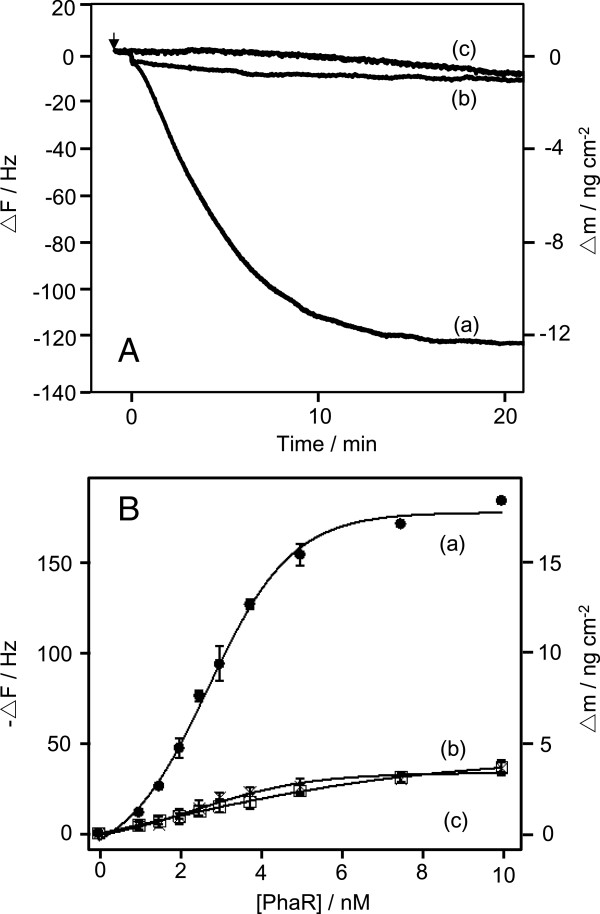
**Binding behavior of PhaR to target DNAs. (A)** Typical time courses of the frequency changes of the *phaP* promoter region (curve a), *phaR* promoter region (curve b), and 0-site DNA immobilized on a QCM oscillator (curve c), in response to the addition of PhaR. The arrow indicates the time of enzyme injection. [PhaR] = 3.75 nM, [DNA] = 19 ± 1 ng (0.55 ± 0.02 pmol) cm^-2^ on a QCM, in 10 mM HEPES buffer solution (pH 7.4) containing 150 mM NaCl and 0.001% Tween 20 at 25°C. **(B)** Saturation binding of PhaR to each sequence of *phaP* promoter region DNA (curve a and ●), *phaR* promoter region DNA (curve b and ×) and 0-site DNA immobilized on to a QCM (curve c and □). Each curve was fitted with a sigmoid curve.

In order to measure the binding behavior of PhaR to 5′-biotinylated dsDNAs (50 bp), the DNA fragments with *phaR*-binding sequences (the promoter regions of *phaR* and *phaP*) and a non-specific sequence (negative control) were immobilized on the electrode of a QCM by biotin-avidin linkage, according to methods outlined in previous papers (Matsuno et al. 2001; Okahata et al. 1998). PhaR was purified using the His-tag purification system, and the purity of PhaR was confirmed by SDS-PAGE (Figure 1). The binding behaviors of PhaR to the DNA fragments were monitored. Figure 2A shows a typical frequency decrease (mass increase) as a function of time, in response to the addition of PhaR. PhaR mainly bound to the DNA containing the *phaP* promoter region (curve a), and barely bound to the DNA containing the *phaR* promoter region (curve b) and the control DNA (curve c). Figure 2B shows that the amount of the bound PhaR (*Δm*) followed a saturation curve as a function of the PhaR concentration ([PhaR]). These binding curves formed a sigmoid curve.

**Figure 3:**
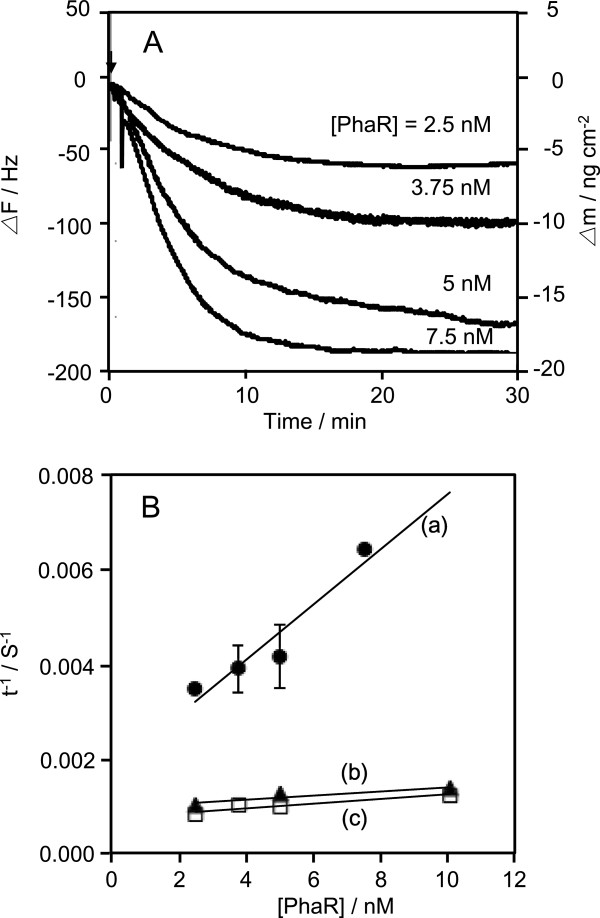
**The influence of PhaR concentrations on the rate of binding to target DNAs. (A)** Binding behaviors of PhaR to the DNA including the *phaP* promoter region in response to changes in PhaR concentration. The arrow indicates the time of enzyme injection. **(B)** Linear reciprocal plots of the relaxation rate (τ^-1^) against the PhaR concentrations according to Eq. (4) in the text.

Next, we attempted to determine the kinetic parameters (*k*_on_, *k*_off_, and *K*_d_ values) of the binding of PhaR to DNAs. The process by which PhaR binds to the DNAs is described by Equation 1 (Okahata et al. 1998). The amount of the DNA/PhaR complex ([DNA/PhaR]) formed after the injection is given by Equations 2 and 3. The fitting curves of the decreases in frequency at various PhaR concentrations gave the relaxation time (τ) and the relaxation rate (τ^-1^) of PhaR binding. When the concentration of PhaR increased from 2.5 to 10 nM, the amount of PhaR bound to the DNA increased (Figure 3A). In addition, the τ value decreased with the concentration of PhaR. The 1/τ value at each PhaR concentration was plotted against the concentrations of PhaR, according to Equation 4 (Figure 3B). The PhaR binding and dissociation rate constants (*k*_on_ and *k*_off_) were obtained from the slope and the intercept of Equation 4, respectively. The *K*_d_ values were obtained from the ratio *k*_off_/*k*_on_. The kinetic parameters, *k*_on_, *k*_off_, *K*_d_, for the target DNAs and control DNA are summarized in Table 1.

(1) $$\begin{array}{c} k_{\mathrm{on}}\\ \mathrm{PhaR}+\mathrm{DNA}⇄\mathrm{PhaR}/\mathrm{DNA}\\ k_{\mathrm{off}} \end{array}$$

(2) $${\left[\mathrm{DNA}/\mathrm{PhaR}\right]}_{t}={\left[\mathrm{DNA}/\mathrm{PhaR}\right]}_{\max}\left\{1-\exp\left(-t/\tau \right)\right\}$$

(3) $$\Delta m_{t}=\Delta m_{\max}\left\{1–\exp\left(-t/\tau \right)\right\}$$

(4) $$1/\tau =k_{\mathrm{on}}\left[\mathrm{PhaR}\right]+k_{\mathrm{off}}$$

**Figure 4:**
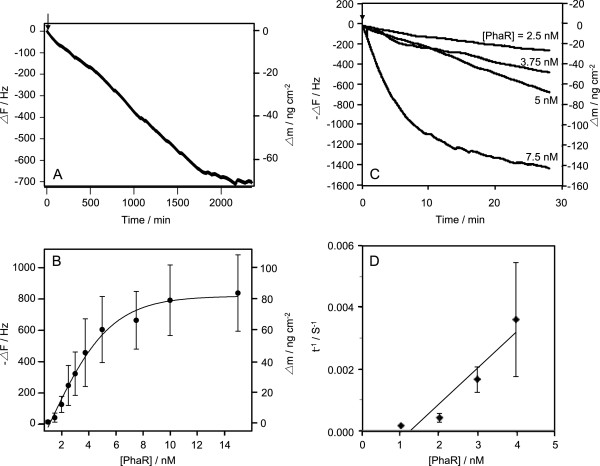
**Binding behavior of PhaR to am-P(3HB) thin films. (A)** Typical time courses of the frequency changes of am-P(3HB) coated on a QCM oscillator, in response to the addition of PhaR. **(B)** Saturation binding of PhaR to P(3HB). The curve was fitted with a sigmoid curve. [P(3HB)] = 200 μg cm^-2^ on a QCM, in 10 mM HEPES buffer solution (pH 7.4), 150 mM NaCl, and 0.001% Tween 20 at 25°C. **(C)** Binding behavior of PhaR to P(3HB) in response to change in the PhaR concentration. The arrow indicates the time of enzyme injection. **(D)** Linear reciprocal plots of the relaxation rate (τ^-1^) against PhaR concentrations according to Eq. (4) in the text.

We also investigated the binding of PhaR to P(3HB) granules in microorganisms using a QCM sensor coated with am-P(3HB) (Figure 4A). This is because the P(3HB) native granules are mainly composed of am-P(3HB). The amount of PhaR bound on am-P(3HB) thin films increased when the concentration of PhaR increased from 1 to 15 nM (Figure 4B and C). Interestingly, as with the binding curve against the target DNA, the binding curve against am-P(3HB) exhibited a sigmoid curve. These results indicated that PhaR bound to P(3HB) in a similar manner as to DNA. The kinetic parameters (*k*_on_ and *k*_off_) were calculated from Equations 1 to 4 (Figure 4D and Table 1). The *k*_on_ value for am-P(3HB) (*k*_on_ = 7.0 ± 3.8 × 10^4^ M^-1^ s^-1^) showed no significant difference from that for DNA containing the *phaP* promoter region (*k*_on_ = 6.0 ± 0.4 × 10^4^ M^-1^ s^-1^).

## Discussion

In order to understand the regulatory system governing PHA production in detail, we investigated the binding behaviors of PhaR to the target DNA (containing the promoter region of *phaP*) and to P(3HB), using QCM measurements. Regarding PhaR-DNA binding, Figure 2A shows that PhaR mainly bound to the DNA containing the *phaP* promoter region (curve a), and barely bound to the DNA containing the *phaR* promoter region (curve b) or to the control DNA (curve c). The binding curve of PhaR to the *phaP* promoter region showed sigmoid curve, implying that PhaR binds to target DNA in a cooperative reaction. The SPR analysis of PhaR-DNA binding in previous studies was not capable of monitoring the initial binding of PhaR, because the concentration of PhaR (10 μM) was higher than in the present experimental conditions (2.5 to 10 nM) (Kojima et al. 2006; Maehara et al. 2002). The higher binding affinity of PhaR to the *phaP* promoter region accorded with the results of gel-mobility-shift assays (Maehara et al. 2002). The DNA fragments with the *phaP* promoter region shifted at a lower concentration of PhaR compared to the DNA fragments that contained the *phaR* promoter region (Potter et al. 2002).

The binding rate constant for the DNA containing the *phaR* promoter region (*k*_on_ = 0.5 × 10^4^ M^-1^ s^-1^) was similar to the parameters for the control DNA (*k*_on_ = 0.4 × 10^4^ M^-1^ s^-1^) (Table 1). Moreover, the dissociation rate constant for the DNA containing the *phaP* promoter region (*k*_off_ = 1.7 ± 0.4 × 10^-3^ s^-1^) was not significantly different from the dissociation rate constant for the DNA containing the *phaR* promoter region (*k*_off_ = 0.9 × 10^-3^ s^-1^) or that of the control DNA (*k*_off_ = 0.7 × 10^-3^ s^-1^). These parameters indicate that PhaR had higher affinity for the *phaP* promoter region than for the *phaR* promoter region. The larger *k*_on_ value for the DNA with the *phaP* promoter region must have been due to the length of the recognition sequence for PhaR in the target DNA region. A 32-bp region TGC-rich sequence is recognized by PhaR in the *phaP* promoter region, while the *phaR* promoter region included only an 8-bp recognition sequence (Table 1) (Potter and Steinbuchel 2005). On the basis of the *k*_on_ values obtained in this study, the difference in *k*_on_ values between the promoter regions of *phaP* and *phaR* corresponds to the hypothetical model of PhaR-mediated *phaP* expression (Maehara et al. 2002; Potter and Steinbuchel 2005; Yamada et al. 2007). In particular, when PHA is not accumulated in the cells, the presence of PhaR is necessary to repress the gene expression of *phaP.* Since PhaP is a predominantly PHA granule-associated protein, PhaP production is not required for the cells without PHA accumulation (Maehara et al. 2002). Thus, the lower *k*_on_ value for DNA with the *phaR* promoter region indicates weak repression of *phaR* expression in cells.

In the measurement of PhaR-am-P(3HB) binding, we did not obtain *k*_off_ and *K*_d_ values of the binding of PhaR to am-P(3HB), because the *k*_off_ was negative. This result indicates that the binding of PhaR to am-P(3HB) is an irreversible interaction (Table 1). There was no significant difference between the *k*_on_ value for am-P(3HB) (*k*_on_ = 7.0 ± 3.8 × 10^4^ M^-1^ s^-1^) and that for the DNA containing the *phaP* promoter region (*k*_on_ = 6.0 ± 0.4 × 10^4^ M^-1^ s^-1^), which implied that the derepression of *phaP* expression was prompted by an increase in the concentration of am-P(3HB) in the cells. In other words, the concentration-dependent effect was one of the main factors initiating the expression of *phaP* at the onset of the dissociation of PhaR from the *phaP* promoter region in cells.

In conclusion, we observed initial binding behaviors between PhaR and target molecules such as target DNAs and am-P(3HB), using QCM techniques. Based on the QCM data, kinetic parameters (*k*_on_, *k*_off_, and *K*_d_) for the binding of PhaR to target molecules were determined by the kinetic analysis of obtained binding curves. These values provided a novel insight into the binding behavior of PhaR with target molecules. The *phaP* gene is likely derepressed in harmony with the ratio of the concentration of the target DNA to the concentration of am-P(3HB) at the beginning of P(3HB) synthesis in microbes. On the basis of the results of a previous paper (Maehara et al. 2002), we assumed that PhaR dissociates from the PhaR/DNA complex when P(3HB) is accumulated under intracellular conditions. This finding indicates that the effector molecules of PhaR are P(3HB) molecules. Also, one of the factors responsible for the dissociation of PhaR from DNA is the high affinity of PhaR to P(3HB). The binding of PhaR to DNA and to am-P(3HB) showed similar *k*_on_ values, suggesting that a concentration-dependent effect caused the expression of *phaP* with dissociation of PhaR from the *phaP* promoter region. The insights of the regulation mechanism concerning PhaR in PHA synthesis have the potential to improve the applications of PHA in white and red biotechnology.

## Abbreviations

(PHA): Polyhydroxyalkanoate; (PhaP): Phasin; [P(3HB)]: Poly(3-hydroxybutyrate); [cr-P(3HB)]: Poly[(*R*)-3-hydroxybutyrate]; (QCM): Quartz crystal microbalance; (*k*_on_): Binding rate constant; (*k*_off_): Dissociation rate constant; (*K*_d_): Dissociation constant; [am-P(3HB)]: Amorphous poly(3-hydroxybutyrate); (SDS): Sodium dodecyl sulfate; (PAGE): Polyacrylamide gel electrophoresis.

## Competing interests

The authors declare that they have no competing interests.

## References

[B1] Backstrom BT Brockelbank JA Rehm BH Recombinant Escherichia coli produces tailor-made biopolyester granules for applications in fluorescence activated cell sorting: functional display of the mouse interleukin-2 and myelin oligodendrocyte glycoprotein BMC Biotechnol 2007 7 3 10.1186/1472-6750-7-3 17204164 PMC1781935

[B2] Banki MR Gerngross TU Wood DW Novel and economical purification of recombinant proteins: intein-mediated protein purification using *in vivo* polyhydroxybutyrate (PHB) matrix association Prot sci publ Prot Soc 2005 14 6 1387 1395 10.1110/ps.041296305 PMC2253394 15883185

[B3] Doi Y Kitamura S Abe H Microbial synthesis and characterization of poly(3-hydroxybutyrate-*co*-3-hydroxyhexanoate) Macromolecules 1995 28 14 4822 4828 10.1021/ma00118a007

[B4] Eugenio LI Galan B Escapa IF Maestro B Sanz JM Garcia JL Prieto MA The PhaD regulator controls the simultaneous expression of the *pha* genes involved in polyhydroxyalkanoate metabolism and turnover in *Pseudomonas putida* KT2442 Environ Microbiol 2010 12 6 1591 1603 10.1111/j.1462-2920.2010.02199.x 20406286

[B5] Kojima T Yamane T Nakano H *In vitro* selection of DNA binding sites for transcription factor, PhaR, from *Paracoccus denitrificans* using genetic library on microbeads and flow cytometry J Biosci Bioeng 2006 101 5 440 444 10.1263/Jbb.101.440 16781475

[B6] Maehara A Taguchi S Nishiyama T Yamane T Doi Y A repressor protein, PhaR, regulates polyhydroxyalkanoate (PHA) synthesis via its direct tnteraction with PHA J Bacteriol 2002 184 14 3992 4002 10.1128/Jb.184.14.3992-4002.2002 12081972 PMC135160

[B7] Matsuno H Niikura K Okahata Y Design and characterization of asparagine- and lysine-containing alanine-based helical peptides that bind selectively to A center dot T base pairs of oligonucleotides immobilized on a 27 MHz quartz crystal microbalance Biochemistry-Us 2001 40 12 3615 3622 10.1021/bi001699o 11297428

[B8] Okahata Y Niikura K Sugiura Y Sawada M Morii T Kinetic studies of sequence-specific binding of GCN4-bZIP peptides to DNA strands immobilized on a 27-MHz quartz-crystal microbalance Biochemistry-Us 1998 37 16 5666 5672 10.1021/bi980037k 9548952

[B9] Potter M Madkour MH Mayer F Steinbuchel A Regulation of phasin expression and polyhydroxyalkanoate (PHA) granule formation in *Ralstonia eutropha* H16 Microbiology 2002 148 2413 2426 12177335 10.1099/00221287-148-8-2413

[B10] Potter M Steinbuchel A Poly(3-hydroxybutyrate) granule-associated proteins: Impacts on poly(3-hydroxybutyrate) synthesis and degradation Biomacromolecules 2005 6 2 552 560 10.1021/Bm049401n 15762612

[B11] Steinbuchel A Fuchtenbusch B Bacterial and other biological systems for polyester production Trend Biotechnol 1998 16 10 419 427 10.1016/S0167-7799(98)01194-9 9807839

[B12] Sudesh K Abe H Doi Y Synthesis, structure and properties of polyhydroxyalkanoates: biological polyesters Prog Polym Sci 2000 25 10 1503 1555 10.1016/S0079-6700(00)00035-6

[B13] Takahashi S Matsuno H Furusawa H Okahata Y Kinetic analyses of divalent cation-dependent *Eco*RV digestions on a DNA-immobilized quartz crystal microbalance Anal Biochem 2007 361 2 210 217 1016/j.ab.2006.11.030 17217909 10.1016/j.ab.2006.11.030

[B14] Takahashi S Matsuno H Furusawa H Okahata Y Direct monitoring of allosteric recognition of type IIE restriction endonuclease EcoRII J Biol Chem 2008 283 22 15023 15030 1074/jbc.M800334200 18367450 10.1074/jbc.M800334200 PMC3258892

[B15] Wang Z Wu H Chen J Zhang J Yao Y Chen GQ A novel self-cleaving phasin tag for purification of recombinant proteins based on hydrophobic polyhydroxyalkanoate nanoparticles Lab on a chip 2008 8 11 1957 1962 10.1039/b807762b 18941699

[B16] Wang ZH Ma P Chen J Zhang J Chen CB Chen GQ A transferable heterogeneous two-hybrid system in Escherichia coli based on polyhydroxyalkanoates synthesis regulatory protein PhaR Microbial cell factories 2011 10 21 10.1186/1475-2859-10-21 21477323 PMC3079617

[B17] Yamada M Yamashita K Wakuda A Ichimura K Maehara A Maeda M Taguchi S Autoregulator protein PhaR for biosynthesis of polyhydroxybutyrate [P(3HB)] possibly has two separate domains that bind to the target DNA and P(3HB): Functional mapping of amino acid residues responsible for DNA binding J Bacteriol 2007 189 3 1118 1127 10.1128/Jb.01550-06 17122335 PMC1797304

[B18] Yamashita K Yamada M Numata K Taguchi S Nonspecific hydrophobic interactions of a repressor protein, PhaR, with poly[(*R*)-3-hydroxybutyrate] film studied with a quartz crystal microbalance Biomacromolecules 2006 7 8 2449 2454 10.1021/Bm060442o 16903695

